# Robotic surgery versus Laparoscopic surgery for anti-reflux and hiatal hernia surgery: a short-term outcomes and cost systematic literature review and meta‐analysis

**DOI:** 10.1007/s00423-024-03368-y

**Published:** 2024-06-06

**Authors:** Diogo Gonçalves-Costa, José Pedro Barbosa, Rodrigo Quesado, Vítor Lopes, José Barbosa

**Affiliations:** 1 Faculty of Medicine, University of Porto, Al. Prof. Hernâni Monteiro, 4200-319 Porto, Portugal; 2 MEDCIDS - Department of Community Medicine, Information and Decision in Health, Faculty of Medicine, University of Porto, Porto, Portugal; 3 Department of Stomatology, São João University Hospital Center, Porto, Portugal; 4 Department of Surgery and Physiology, Faculty of Medicine, University of Porto, Porto, Portugal; 5 Department of General Surgery, São João University Hospital Center, Porto, Portugal

**Keywords:** Hiatal Hernia, Robotic, Laparoscopic, Outcomes, Cost

## Abstract

**Purpose:**

The objective of this study is to compare the operative time, intraoperative complications, length of stay, readmission rates, overall complications, mortality, and cost associated with Robotic Surgery (RS) and Laparascopic Surgery (LS) in anti-reflux and hiatal hernia surgery.

**Methods:**

A comprehensive literature search was conducted using MEDLINE (via PubMed), Web of Science and Scopus databases. Studies comparing short-term outcomes and cost between RS and LS in patients with anti-reflux and hiatal hernia were included. Data on operative time, complications, length of stay, readmission rates, overall complications, mortality, and cost were extracted. Quality assessment of the included studies was performed using the MINORS scale.

**Results:**

Fourteen retrospective observational studies involving a total of 555,368 participants were included in the meta-analysis. The results showed no statistically significant difference in operative time, intraoperative complications, length of stay, readmission rates, overall complications, and mortality between RS and LS. However, LS was associated with lower costs compared to RS.

**Conclusion:**

This systematic review and meta-analysis demonstrates that RS has non-inferior short-term outcomes in anti-reflux and hiatal hernia surgery, compared to LS. LS is more cost-effective, but RS offers potential benefits such as improved visualization and enhanced surgical techniques. Further research, including randomized controlled trials and long-term outcome studies, is needed to validate and refine these findings.

**Supplementary Information:**

The online version contains supplementary material available at 10.1007/s00423-024-03368-y.

## Introduction

The surgical landscape for Gastroesophageal Reflux Disease (GERD) and hiatal hernias has evolved significantly, transitioning from traditional open approaches to minimally invasive techniques. Laparoscopic surgery (LS) has become the standard of care, offering reduced postoperative discomfort, shorter hospital stays, and quicker recovery, making it a preferred choice in the surgical community. However, the technical demands of LS, particularly in complex hiatal hernia repairs, require a high level of surgical expertise.

Robotic-assisted Surgery (RS) represents a major advancement in minimally invasive surgery. With enhanced three-dimensional visualization, improved dexterity, and ergonomic advantages, RS potentially overcomes some limitations of conventional laparoscopy. These features are beneficial in the precise dissection and suturing required for anti-reflux and hiatal hernia procedures. Despite these advantages, the broader adoption of RS is moderated by ongoing debates regarding its cost-effectiveness and the current level of evidence, which has yet to definitively establish its superiority over LS in terms of clinical outcomes.

This study aims to conduct a rigorous comparison of RS and LS in the context of anti-reflux and hiatal hernia surgery, focusing on operative outcomes and economic considerations. By synthesizing data from recent studies and observational analyses, this analysis seeks to provide comprehensive insights for surgeons in choosing the most suitable surgical approach, considering the balance between technological advancements and practical aspects of patient safety, surgical efficacy, and healthcare economics.

## Materials and methods

This systematic review and meta-analysis was reported according to the preferred reporting items for systematic reviews and meta-analysis (PRISMA) guidelines [29].

### Literature search strategy

A systematic review of literature was performed by two authors independently using the MEDLINE (via PubMed), Web of Science and Scopus databases. A comprehensive literature search was performed on September 6th 2023. The query “(robot*) AND (lapar*) AND (cost) AND (((hernia) AND (hiat*)) OR (fundoplic*) OR (GERD) OR (anti-reflux)) AND (y_10[Filter])” was used for the PubMed database search. For the Web of Science search was used the following query: “(robot*) AND (lapar*) AND (cost) AND (((hernia) AND (hiat*)) OR (fundoplic*) OR (GERD) OR (anti-reflux)) [Topic]” with a 10 year and (Article or Review article) filter. The search strategy used in Scopus was as follows: “ TITLE-ABS-KEY ( ( robot*) AND ( lapar*) AND ( cost) AND ( ( ( hernia) AND ( hiat*)) OR ( fundoplic*) OR ( gerd) OR ( anti-reflux))) AND PUBYEAR > 2012 AND PUBYEAR < 2024 AND ( LIMIT-TO ( SUBJAREA, "MEDI")) AND ( LIMIT-TO ( DOCTYPE, "ar") OR LIMIT-TO ( DOCTYPE, "re")) AND ( LIMIT-TO ( LANGUAGE, "English")) AND ( EXCLUDE ( EXACTKEYWORD, "Child"))”.

### Study selection and eligibility criteria

We included observational clinical studies that compared short-term outcomes and cost comparison between the two surgical approaches (RS and LS), in patients with GERD and/or hiatal hernia, who underwent curative-intent surgery.

The search was restricted to studies written in English, from January 2013 until September 2023. The time frame selected for our study was intentionally chosen to reflect two primary considerations: the maturation of surgical expertise in both robotic and laparoscopic techniques and the representation of current patient demographics and comorbidities [31]. While RS has been in practice since early 2000’s, it is a relatively newer field compared to laparoscopic surgery [12]. The proficiency and widespread adoption of robotic surgery have notably increased in recent years. By selecting a more contemporary time window, our study aims to compare both techniques during a period where the surgical community possesses a more balanced and matured expertise in each, thus providing a fair comparison of their effectiveness and outcomes.

Studies of pediatric age patients, animal studies, conference abstract, comments, reviews, and guidelines were excluded.

**Fig. 1:**
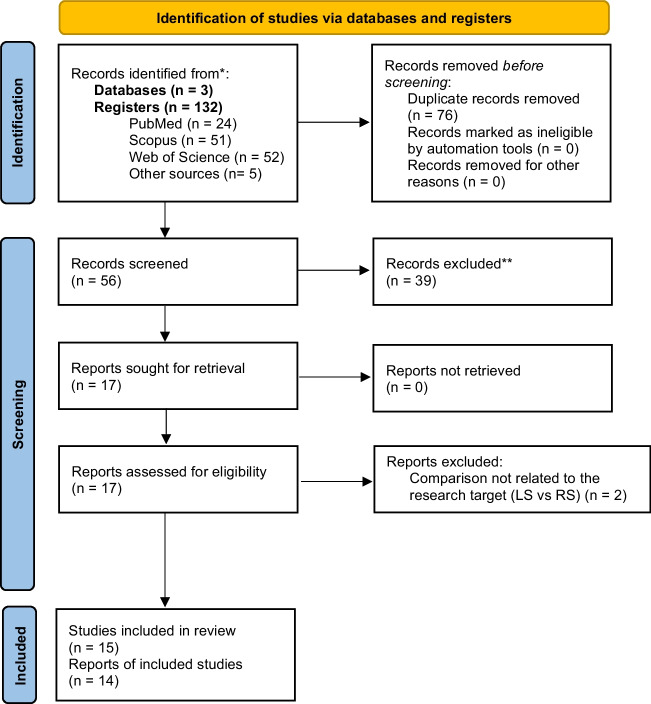
PRISMA Flowchart

First, relevant studies were selected based on their titles and abstracts. Then, a more thorough selection process was conducted by reading the full articles. The inclusion and exclusion criteria, as defined beforehand, were used to guide this selection. Additionally, any articles referenced in the selected studies and considered relevant to the study were also included. Figure 1 demonstrates the process in detail.

### Data extraction

Data extraction was performed independently by two authors and compared at the end of the reviewing process, with disputes being resolved by a third author. The following data was extracted: author, year of publication, the study design, sample size, patients’ characteristics (sex, age, body mass index (BMI)), hiatal hernia type, surgical approach, operative time and costs, intra-operative complications, conversion to open, length of stay (LOS), readmission rates, overall complications, and mortality.

### Quality assessment

To proceed with the quality assessment of these studies, the Methodological Index for Non-Randomized Studies (MINORS) checklist [34], was used by two independent reviewers. The Methodological Index for Non-Randomized Studies (MINORS) is a validated instrument designed to assess the quality of non-randomized surgical studies, both comparative and non-comparative. Developed by Slim et al. [34] in 2003, MINORS provides a systematic approach for evaluating the methodological quality of this type of research, which is inherently prone to bias due to the lack of randomization. The MINORS index consists of 12 items for comparative studies and 8 for non-comparative studies. Each item is scored on a scale of 0 to 2, where 0 indicates that the criterion is not reported, 1 suggests that the criterion is reported but inadequate, and 2 signifies that the criterion is reported and adequate.

### Statistical analysis

We conducted the meta-analysis using Review Manager (Version 5.4.1). For dichotomous outcomes, we reported the results as odds ratios (OR) with 95% confidence intervals (CIs), calculated using the Mantel–Haenszel method. For continuous outcomes, we reported the results as mean differences (MD) with 95% CI, calculated using the generic inverse variance method. Some studies presented their outcomes as median and range, so we used a method described by Wan et al. [36] to estimate the mean and standard deviation. We considered a significance level (α) of 0.05. To assess heterogeneity, we used the Chi-squared (χ2) test and the I-squared (I^2^) measure. Given the clinical heterogeneity of the included studies, we applied a random effects model. Heterogeneity was classified as low, moderate, and high when I^2^ was greater than 25%, 50%, and 75%, respectively. We assessed the existence of publication bias among included studies using funnel plots, provided as Supplementary Material 1.

## Results

### Search results and characteristics of the included studies

The initial search of the MEDLINE (via PubMed), Web of Science and Scopus platforms resulted in 24, 52, and 51 studies, respectively, totaling 127 potentially relevant articles. Out of the total, 76 duplicates were excluded. After reviewing the titles and abstracts, an additional 39 articles were excluded, leaving 17 full-text articles for analysis. Of these, 14 studies that met the eligibility criteria for qualitative and quantitative analysis were obtained. Five studies from a recent meta-analysis, that contained some studies not captured by the designated queries, were also included [22]. Figure 1 presents a flowchart illustrating the reasons for excluding the remaining articles at each step of the process (Table 1).

**Table 1:** MINORS

Author	Year	1)	2)	3)	4)	5)	6)	7)	8)	9)	10)	11)	12)	Total
Gehrig et al.	2013	2	2	1	2	2	2	2	0	2	2	0	2	19
Owen et al.	2014	2	2	1	2	1	2	2	0	2	2	1	2	19
Wormer et al.	2014	2	2	1	1	2	2	2	0	2	2	1	2	19
Higgins et al.	2017	2	2	1	1	2	2	2	0	2	2	1	1	18
Howell et al.	2020	2	2	1	2	2	2	2	0	2	2	1	1	19
Soliman et al.	2020	2	2	2	2	2	2	2	0	2	2	0	2	20
Hosein et al.	2021	2	2	1	2	2	2	2	0	2	2	1	2	20
Benedix et al.	2021	2	2	2	2	2	2	2	0	2	2	2	2	22
Gerull et al.	2021	2	2	2	2	2	2	2	0	2	2	2	2	22
Wilhelm et al.	2022	2	2	2	2	2	2	2	0	2	2	2	2	22
Lekarczyk et al.	2022	2	2	2	2	2	2	2	0	2	2	1	2	21
Napolitano et al.	2022	2	2	1	2	1	2	2	0	2	2	1	2	19
Klock et al.	2023	2	2	1	1	2	2	2	0	2	2	1	2	19
Munshower et al.	2023	2	2	1	2	2	2	2	0	2	2	2	2	21

Values 0: not reported; 1: reported but inadequate; 2: reported and adequate

1) a clearly stated aim; 2) inclusion of consecutive patients; 3) prospective collection of data; 4) end points appropriate to the aim of the study; 5) unbiased assessment of study end point; 6) follow-up period appropriate to the aim of the study; 7) loss of follow-up less than 5%; 8) prospective calculation of the study size; 9) an adequate control group; 10) contemporary groups; 11) baseline equivalence groups; 12) adequate statistical analyses

Overall, these 14 studies involved a total of 555,368 participants, with 66,725 undergoing RS and 488,643 undergoing LS. All of the studies were retrospective [5, 7, 8, 13–15, 18, 26–28, 35, 38] observational studies, except for two [19, 37] that were conducted in a prospectively manner. Detailed information on study characteristics and details of the patients was presented in Supplementary Material 2. When available, further characterization of the patients was done based on hernia type (Table 2) and surgery characteristics (type of fundoplication, use of mesh and Collis procedure) (Table 3).

**Table 2:** Types of Hiatal Hernia

	Size of Hiatal Hernia (N)		Type of Hiatal Hernia (N)							
	LS	RS	I		II		III		IV	
Author			LS	RS	LS	RS	LS	RS	LS	RS
Gehrig	NR	NR	NR	NR	NR	NR	NR	NR	NR	NR
Owen	NR	NR	NR	NR	NR	NR	NR	NR	NR	NR
Wormer	NR	NR	NR	NR	NR	NR	NR	NR	NR	NR
Higgins	NR	NR	NR	NR	NR	NR	NR	NR	NR	NR
Howell	NR	NR	3	2	7	4	68	36	1	1
Soliman	NR	NR	37	50	0	0	109	87	5	5
Benedix	< 5 cm: (47); 5–10 cm: (13); > 10 cm: (25)	< 5 cm: (28); 5–10 cm: (17); > 10 cm: (10)	NR	NR	NR	NR	NR	NR	NR	NR
Gerull	NR	NR	0	0	12	15	845	659	167	156
Hosein	NR	NR	NR	NR	NR	NR	NR	NR	NR	NR
Wilhelm	NR	NR	4	4	8	2	29	23	1	2
Lekarczyk	NR	NR	4	4	8	2	29	23	1	2
Napolitano	NR	NR	NR	NR	NR	NR	NR	NR	NR	NR
Klock	NR	NR	NR	NR	NR	NR	NR	NR	NR	NR
Munshower	NR	NR	NR	NR	NR	NR	NR	NR	NR	NR

**Table 3:** Surgery characteristics

	Nissen (N)	Toupet (N)	Dor (N)	LINX (N)	Conversion to open (N)	Esophageal lengthnening (Collis)	Mesh used
Author	Laparoscopic Surgery (LS)						
Gehrig	9	NR	NR	NR	1	NR	7
Owen	NR	NR	NR	NR	NR	NR	NR
Wormer	NR	NR	NR	NR	NR	NR	NR
Higgins	NR	NR	NR	NR	NR	NR	6
Howell	22	46	3	0	0	NR	NR
Soliman	23	126	2	0	0	NR	2
Benedix	0	85	0	0	2	NR	55
Gerull	NR	NR	NR	NR	72	113	NR
Hosein	NR	NR	NR		NR	NR	NR
Wilhelm	4	15	0	0	0	NR	10
Lekarczyk	NR	NR	NR	NR	NR	NR	NR
Napolitano	NR	NR	NR	NR	NR	NR	NR
Klock	NR	NR	NR	NR	NR	NR	NR
Munshower	NR	NR	NR	NR	NR	NR	66
Author	Robotic Surgery (RS)						
Gehrig	6	NR	NR	NR	0	NR	5
Owen	NR	NR	NR	NR	NR	NR	NR
Wormer	NR	NR	NR	NR	NR	NR	NR
Higgins	NR	NR	NR	NR	NR	NR	5
Howell	14	22	1	NR	0	NR	24
Soliman	26	63	1	52	1	NR	2
Benedix	55	0	0	NR	1	NR	30
Gerull	NR	NR	NR	NR	0	1	NR
Hosein	NR	NR	NR	NR	NR	NR	NR
Wilhelm	0	29	0	NR	0	NR	36
Lekarczyk	NR	NR	NR	NR	NR	NR	NR
Napolitano	NR	NR	NR	NR	NR	NR	NR
Klock	NR	NR	NR	NR	NR	NR	NR
Munshower	NR	NR	NR	NR	NR	NR	44

### Quality assessment

The median score in the MINORS scale was 19.5, with a range of 18–22, as detailed in Table 1. As the lowest scoring study was a score of 18, all the included studies were deemed suitable for inclusion in the quantitative analysis.

### Operative time

**Fig. 2:**
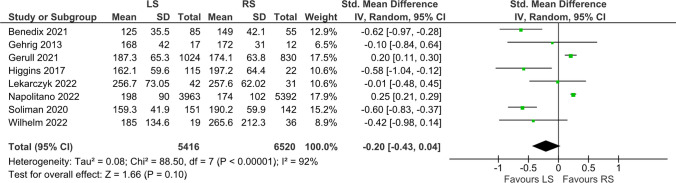
Operative Time

Eight studies, involving a total of 11,936 patients, reported the operative time. [5, 7, 8, 13, 19, 27, 35, 37] There was no statistically significant difference between the LS group and the RS group in terms of operative time. (MD = -0.20, [-0.43, 0.04], p = 0.10) (Fig. 2).

### Intraoperative complications

Given that intra-operative complications were documented in only a minor subset of studies, encompassing merely 0.02% of patients undergoing Laparoscopic Surgery (LS) and 2% of those undergoing Robotic Surgery (RS), we elected to exclude this variable from our meta-analysis. This decision was informed by the concern that the limited and unbalanced reporting could introduce significant bias into our findings.

### Length of Stay (LOS)

**Fig. 3:**
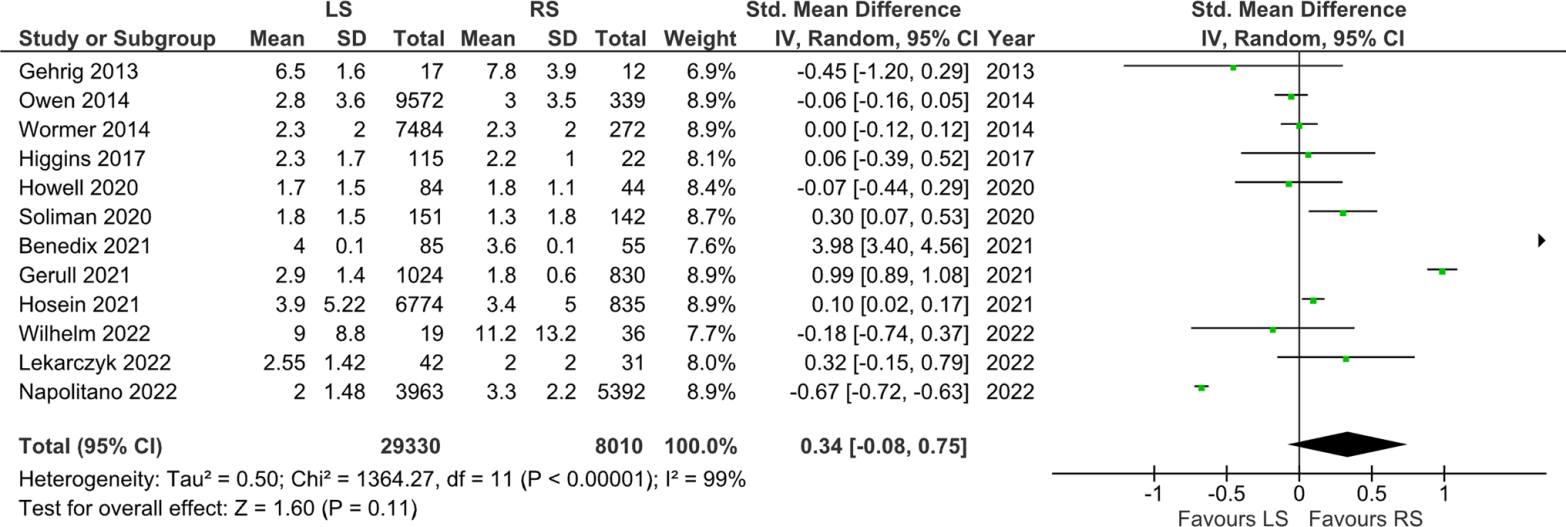
Length of Stay

This outcome was reported in 12 studies. [5, 7, 8, 13–15, 19, 27, 28, 35, 37, 38] The mean length of hospital stay was 3.7 days in the RS group and 3.5 days in the LS group. There was no statistically significant difference (MD = 0.34, [-0.08, 0.75], p = 0.11) (Fig. 3).

### Readmission rate

**Fig. 4:**
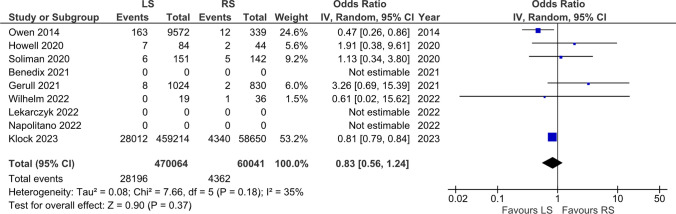
30-day readmissions

A total of 539673 patients from 9 studies were included in the analysis of readmission rates. The results showed no statistically significant difference in readmission between the RS group and the LS group (5.97% vs 6.87%, respectively) (OR = 0.91 [0.67, 1.23] p = 0.53) (Fig. 4).

### Overall postoperative complications

**Fig. 5:**
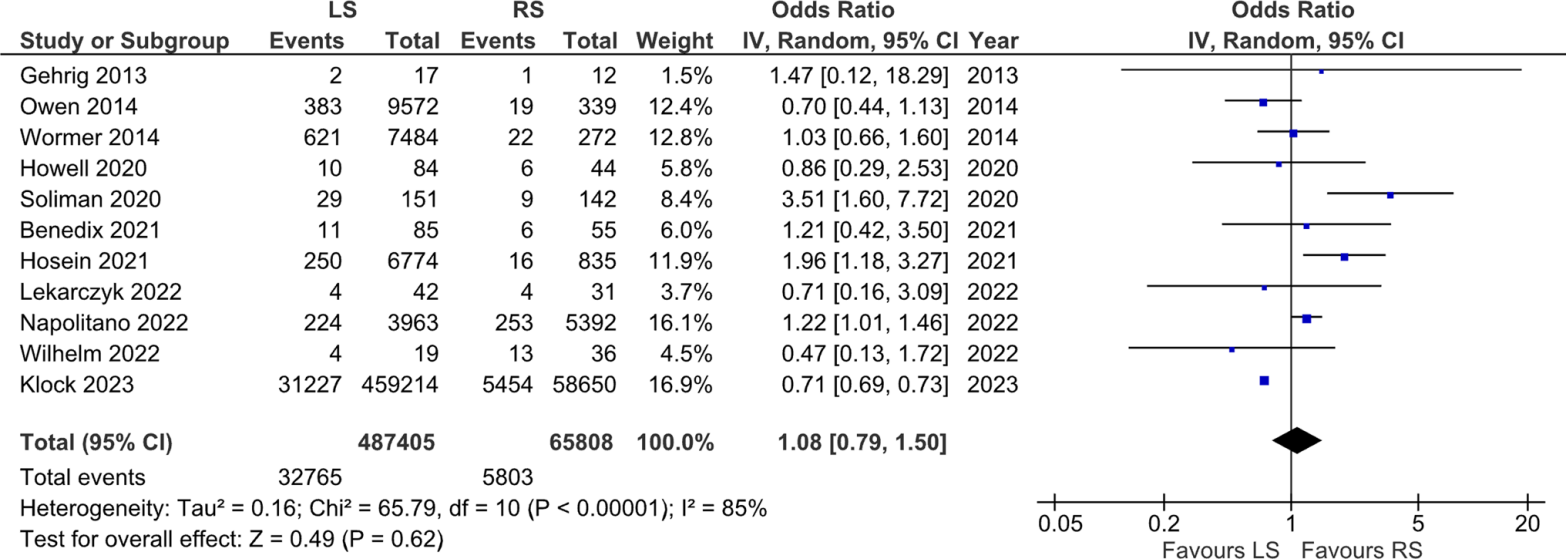
Overall Postoperative Complications

A total of 11 studies were included in the analysis of overall postoperative complications. The results showed no statistically significant difference in postoperative complications between the RS group and the LS group (OR = 1.08, [0.79, 1.50], p = 0.62) (Fig. 5).

### Mortality

**Fig. 6:**
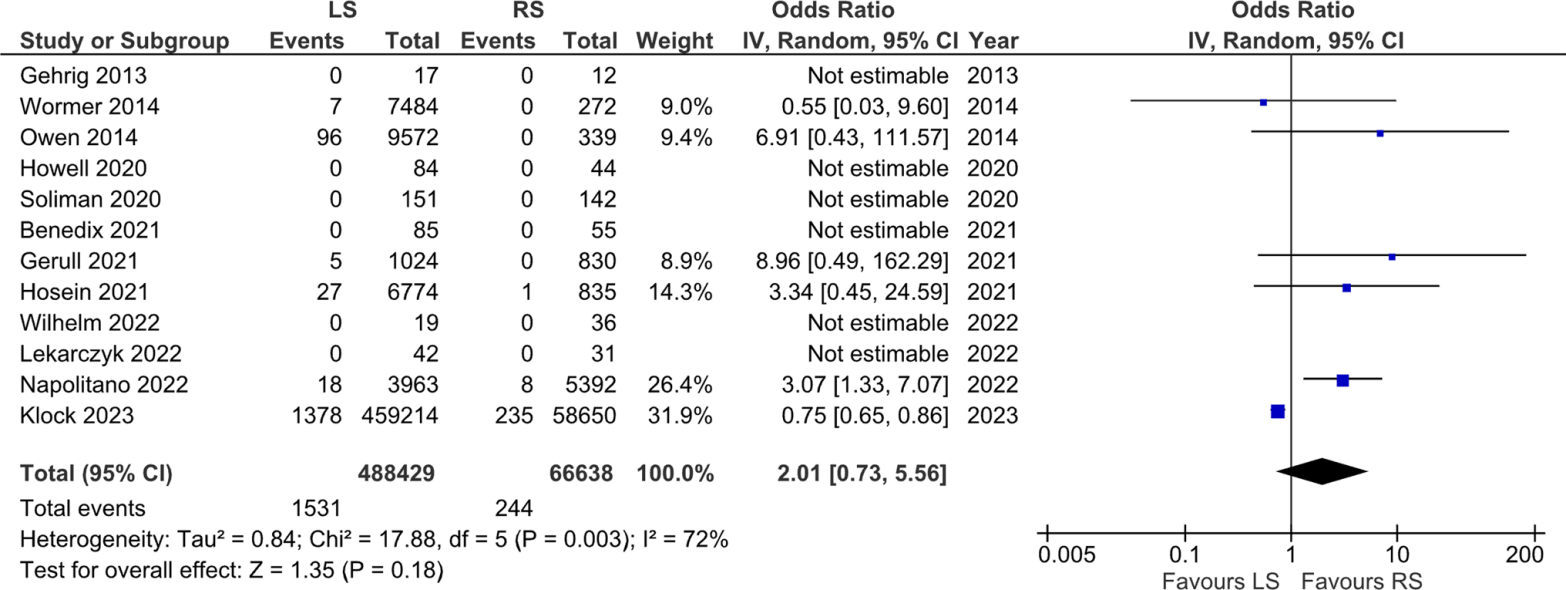
Mortality

Twelve studies were included, and mortality was comparable between both groups (OR = 2.01, [0.73, 5.56], p = 0.18). Mortality rate was 0,4% (244/ 66,638) in the RS group and 0,3% (1531/488429) in the LS group (Fig. 6).

### Cost

**Fig. 7:**
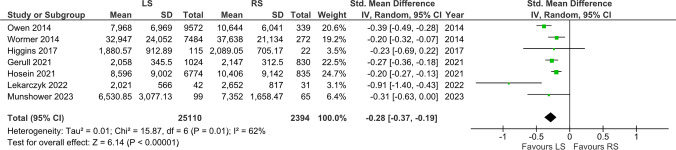
Cost

Seven studies included cost reports and were, therefore, included. The LS group had a statistically significant lower cost (MD = -0.28, [-0.37, -0.19], p < 0.00001) (Fig. 7).

## Discussion

In this systematic literature review and meta-analysis, we compared robotic surgery (RS) to laparoscopic surgery (LS) in the context of anti-reflux and hiatal hernia repair procedures. What has been observed is an increased implementation of robotic surgery in reflux disease, even though advantages of this approach have not been established when compared to laparoscopy [33]. Furthermore, it is acknowledged that when comparing the advantages of laparoscopic surgery with open surgery, laparoscopy is advantageous in the short term, with long-term outcomes being like those of open surgery [11]. In this context, it is crucial to understand the short-term outcomes between a recognized advantageous approach (LS) and a newer one that has been increasingly utilized (RS). Our results show that there was no statistically significant difference in operative time between robotic surgery (RS) and laparoscopic surgery (LS). This finding suggests that, from an operative time perspective, RS is comparable to LS. The slight non-significant difference in favor of LS may be attributed to factors such as surgeon experience and the learning curve associated with RS. Furthermore, an important aspect to consider is the docking time for robotic systems. As technology advances, there is potential for the docking process to become more efficient, potentially reducing overall operative time. This enhancement could further narrow the gap in operative times between RS and LS. As more surgeons become proficient in robotic techniques and as the efficiency of these systems improves, operative times may become even more comparable [20, 30].

The analysis of LOS revealed no statistically significant difference between RS and LS. However, a high level of heterogeneity was observed, suggesting substantial variability among the studies. This variability may be due to factors not accounted for in this analysis, such as patient-specific variables and hospital protocols [6]. The overall weighted mean length of stay (LOS) was around 2.8 days for the LS groups and 3.1 days, which is in-line with previous meta-analysis [21]. While most of the analyzed studies report a shorter LOS, there are exceptions, such as in the studies by Gehrig et al. [7], Benedix et al. [5], and Wilhelm et al. [37]. These studies account for a smaller patient sample (17, 85, and 19 for the LS group, respectively) and exhibit a higher LOS (6.5, 4, and 9 days for the LS group, respectively). The increased LOS in the first two studies is present for both LS and RS groups may reflect center-specific discharge protocols that necessitate longer stays. Wilhelm et al. focuses on a unique patient cohort with complete upside-down stomach (cUDS), a complex and extensive repair that likely demands more rigorous postoperative monitoring before hospital discharge.

Our analysis found no significant difference in 30-day readmission rates between RS and LS, despite a moderate level of statistical heterogeneity. Napolitano et al. [27], Benedix et al. [5] (90 days-readmission reported only) and Lekarczyk et al. [19] (no period declaration) were not included in the meta-analysis. The similarity in readmission rates is a reassuring result, indicating that patients undergoing either procedure have comparable postoperative experiences and outcomes. The study by Owen et al. identifies gastrointestinal issues, including abdominal pain, diarrhea, dysphagia, esophageal reflux, nausea, and vomiting, as the primary reasons for re-admission after laparoscopic surgery. In contrast, for RS, respiratory complications, particularly those associated with lung transplants, were the predominant cause of re-admission. The data analysis indicates a bias: centers that use RS on patients also undergoing lung transplants skew the results. Excluding these cases, re-admission rates for RS and LS are comparable [28]. Klock et al. refers the main for readmission included esophagitis, followed by digestive system diagnoses, electrolyte imbalance, and subsequent procedure to the stomach, esophagus, or duodenum [18]. Wilhelm et al. reports one patient from the RS group was readmitted due to dysphagia and diarrhea after an otherwise uneventful hospital course and discharge. The issues were addressed with further dietary guidance. Notably, there were no deaths within 30 days after surgery in either group [37]. Soliman et al. [35],Gerull et al. [9], Howell et al. [15] did not specify each readmission cause.

The absence of a statistically significant difference in overall postoperative complications between RS and LS further underscores the safety of both surgical technologies. High heterogeneity was detected, possibly due to variations in how complications were defined and reported in the included studies. As standardization in reporting practices improves, as only 3 studies included Clavien-Dindo scores, we may gain a more precise understanding of the comparative safety of RS and LS [17].

Benedix et al. report that LS had a slightly higher rate of postoperative complications (12.9%) compared to RS (10.9%). Dysphagia was the most common adverse event in both groups, with similar frequencies (9.4% in LS vs. 9.1% in RS). The occurrence of pleural effusion was more frequent in the RS group (7.3% vs. 2.6% in LS). Neither group reported postoperative pneumonia, cardiac, or wound complications, and there was no mortality reported. Soliman et al. contrasts these findings by demonstrating a significantly lower complication rate in RS (6.3%) when compared with LS (19.2%). This study highlighted pulmonary complications as a particular concern, being more prevalent in LS patients (7.9%) compared to RS patients (1.4%).

Klock et al. and Napolitano et al. demonstrate a pattern where RS is associated with a different complication profile. Klock et al. reported a 28% higher risk of complications in RS, with notable issues including a higher risk of infection and esophageal perforation, but a lower risk of bleeding compared to LS. Napolitano's findings complement this by showing a higher rate of superficial surgical site infections in RS (1.0% vs. 0.6% in LS), despite lower odds of pulmonary complications and renal failure. Gehrig et al. showcases RS with an 8.3% complication rate compared to 11.8% in LS. Noteworthy is the specific mention of a postoperative bleeding case in RS requiring laparotomy and an incidence of pneumonia prolonging hospital stay.

Howell et al. reports that within the LS group (n = 84) there were 10 cases (11.9%) of postoperative complications. In contrast, the RS group (n = 44) experienced 6 cases (13.6%) of complications. The study also suggests a trend where the use of mesh is associated with an increased incidence of surgical complications. Specifically, the study indicates a higher complication rate in the LS group when mesh is used (18.6%) compared to when no mesh is utilized (8.7%). Wilhelm et al. describes no major postoperative complications of severity level ≥ IIIb in either group, according to the Clavien-Dindo classification. However, minor complications (Clavien-Dindo levels I-IIIa) were more frequent in the RS group (36% vs. 21% in LS, non-statistically significant difference, p = 0.36), with incidents as pleural effusion and pneumothorax.

Owen et al. reports a morbidity of 4% in the LS group (n = 9572) and 5,6% in the RS group (n = 339), while Lekarczyk et al. reports 4 complications in the LS group (n = 42) and 4 in the RS group (n = 31), both studies did not specify. Gerull et al., Munshower et al., Higgins et al., Wormer et al., and Hosein et al. did not provide detailed or stratified complication data, representing a gap in the available information.

Mortality rates had no statistically significant difference observed between the two groups. This finding is consistent with the generally low mortality rates associated with anti-reflux and hiatal hernia procedures [32]. The mortality rate for laparoscopic anti-reflux surgery is reported to be as high as 0.03% [23]. However, it is important to note that most studies we analyzed predominantly report on overall mortality rates. These findings represent the data we have managed to extract and which are documented in the articles. When considering the overall mortality, it is identified that it can escalate to 0.08%, which includes not only the mortality directly linked to the surgical procedure but also other contributing factors. It is crucial to consider that these statistics may involve high-risk cohorts, such as the study by Napolitano et al., which includes U.S. veterans. This population is at an elevated risk due to higher levels of comorbidities compared to the non-veteran population, including higher rates of posttraumatic stress disorder, depression, and cardiovascular disease [1, 4]. Such factors underscore the complexity of assessing mortality rates and highlight the need to consider the specific characteristics of patient cohorts in these studies.

In our study, we conducted a cost evaluation of LS versus RS, which revealed LS as less costly. However, the interpretation of these cost findings is nuanced due to variations in cost reporting across different studies. Previous studies suggest a cost range of 1,534 to 2,257 euros for RS and 657 to 763 euros for LS [10]. A significant methodological consideration is the occasional omission or non-explicit reporting of cost details within these studies, which poses a challenge in the accurate comparison of mean costs. Such inconsistencies, including factors like the utilization of mesh in hernia repairs [26], contribute to a moderate level of statistical heterogeneity observed in the cost data. Given the varied cost structures across healthcare systems, we advise a careful interpretation of these cost findings. As Munshower et al. points out, the cost is specific to each institution because hospital contracts differ across various hospital systems. Hence, other hospitals are advised to compare their own cost data to achieve more relevant and meaningful comparisons [26]. Our goal in including this cost evaluation was to provide a holistic understanding, recognizing the necessity for cautious consideration of the results [16].

In this research, while some of the included studies reported the types of hiatal hernias in the preoperative characteristics of patients, there was no stratification based on the type of hiatal hernia for various outcomes such as intraoperative and postoperative complications, operation duration, LOS, etc. This lack of stratification was also evident for other patient characteristics and surgery characteristics. Future studies should aim to compare robotic surgery with laparoscopic surgery with a clear stratification for each hiatal hernia type. Another notable limitation of our systematic review is the significant weight of the Klock et al. and Owen et al. studies within our analysis pool. Given its substantial sample its absence in certain outcomes markedly diminishes the number of observations and, consequently, the robustness of our findings for that aspect of antireflux procedures.

Additionally, the reliance on observational retrospective studies without randomization leads to uneven cohort distribution between LS and RS, potentially introducing selection bias that may favor either group, and contributing to greater heterogeneity. To obtain more conclusive evidence, there is a critical need for prospective studies and randomized clinical trials to deepen our understanding of the subject. Analyzing certain funnel plots is challenging due to the limited number of studies in some meta-analyses, which might indicate a degree of publication bias, as suggested in the LOS funnel plot (see Supplementary Material 1). Moreover, the inability to access long-term outcomes and symptoms highlights a gap in our current knowledge, presenting an opportunity for future research to explore these aspects more thoroughly. Despite these limitations, our work has the strength of enhancing our meta-analysis by including studies that were previously overlooked or unpublished, thereby expanding the range and depth of the existing analyses [22].

The results of this analysis suggest that, in the short term, RS is non-inferior to LS in terms of surgical outcomes, except for cost. These findings align with previous the studies, such as Markar et al. [24]. LS is less costly, which may be an important consideration for healthcare institutions and governing bodies and payers. However, it is important to consider the broader context of surgical innovation. Robotic surgery offers unique advantages, including improved kinesthetic, miniaturization and microrobotics, enhanced visual feedback with greater magnification and higher fidelity in detail [2], potential for artificial intelligence synergies [3], and ergonomic benefits for surgeons [25]. As RS becomes more widely adopted and surgeons gain experience with the technology, it is reasonable to anticipate that some of the cost differentials may decrease. Advances in robotic technology and increased competition in the market may lead to cost reductions.

## Conclusion

This study indicates that RS holds promise in anti-reflux and hiatal hernia surgery, with short-term outcomes non-inferior to LS. Although cost is currently a limiting factor, we postulate that with increased adoption and technological advancements, the cost-effectiveness of RS may improve, making it a compelling choice for the future. The advantages of RS—including enhanced imaging, AI integration, and ergonomic benefits—should not be overlooked as they contribute to the overall value proposition of this innovative surgical approach. Further research is needed to validate these findings. This should include well-designed randomized controlled trials (RCTs) to verify the results and enhance their reliability. Additionally, cost-effectiveness analyses and long-term outcome studies are necessary to further validate the findings.

### Supplementary Information

Below is the link to the electronic supplementary material.

Supplementary file1 (DOCX 1016 KB)

Supplementary file2 (XLSX 20 KB)

## Data Availability

No datasets were generated or analysed during the current study.
