# Supplementary material for: Robotic surgery versus Laparoscopic surgery for anti-reflux and hiatal hernia surgery: a short-term outcomes and cost systematic literature review and meta‐analysis
Source: Langenbecks Arch Surg. 2024 Jun 6;409(1):175. doi: 10.1007/s00423-024-03368-y (PMC11156741; doi:10.1007/s00423-024-03368-y)
Supplement: Supplementary file 1 — Supplementary file1 (DOCX 1016 KB) [file 423_2024_3368_MOESM1_ESM.docx]

Publication Bias Assessment – Funnel Plots


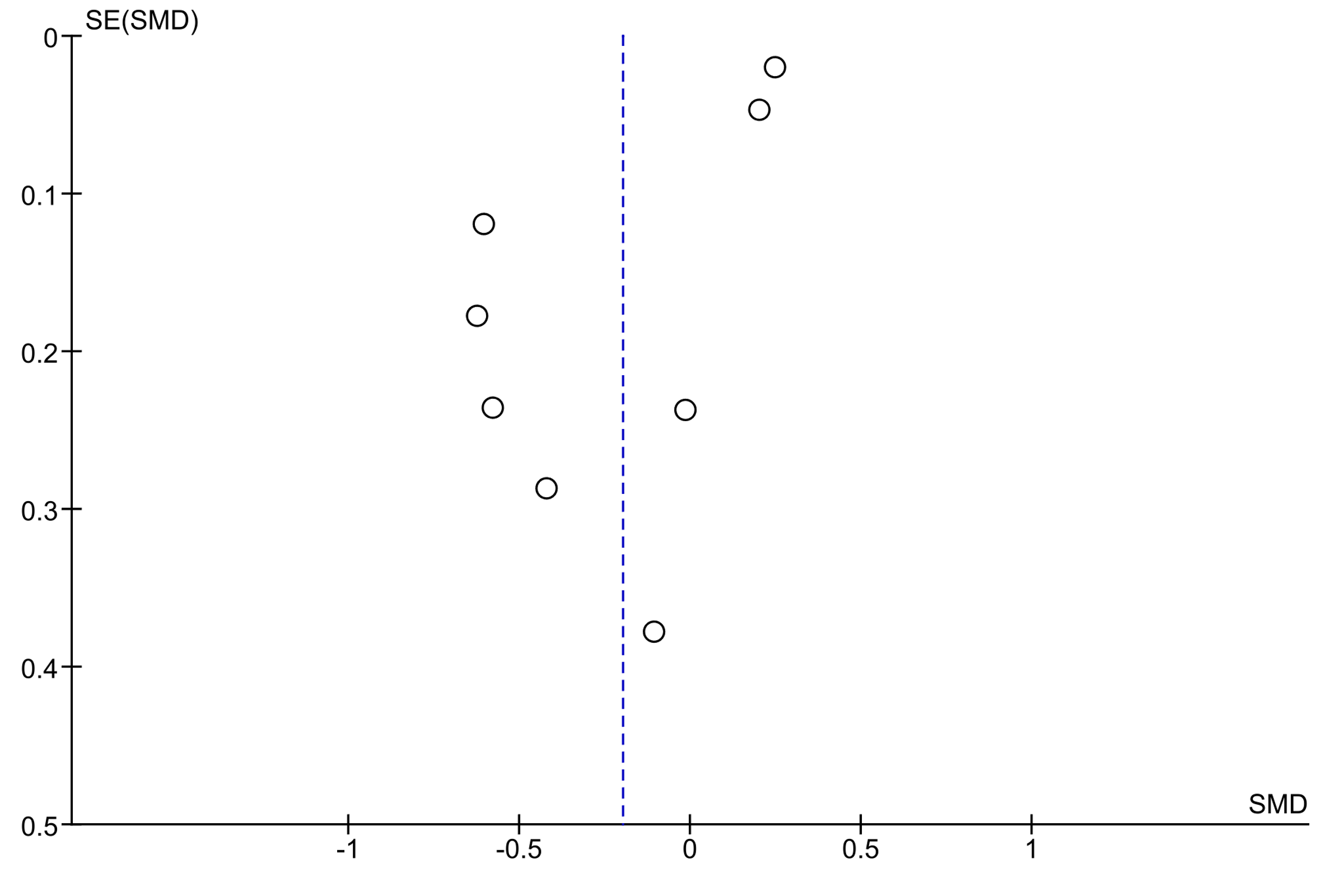


Figure 1- Operative Time


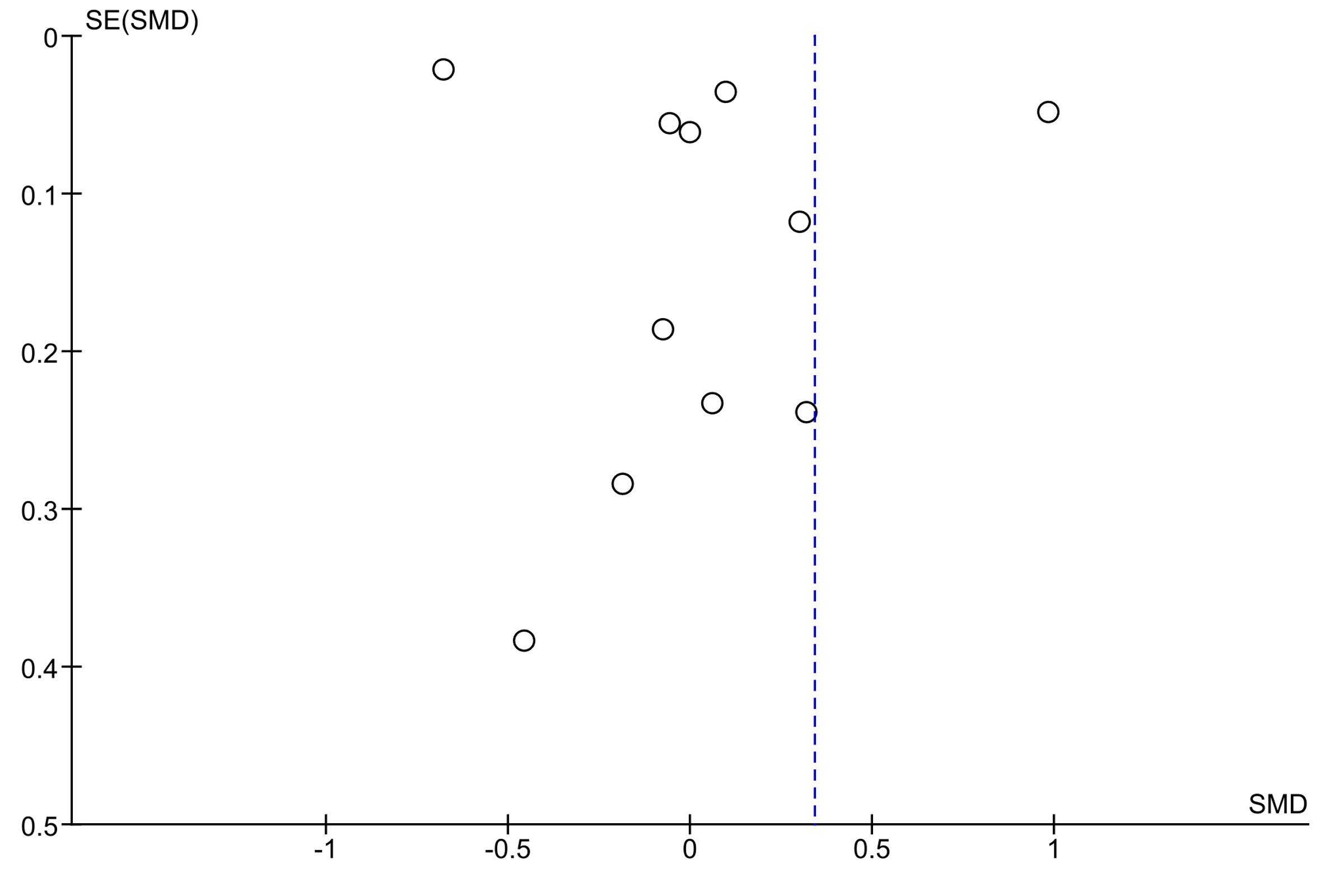


Figure 2 - Length of Stay (LOS)


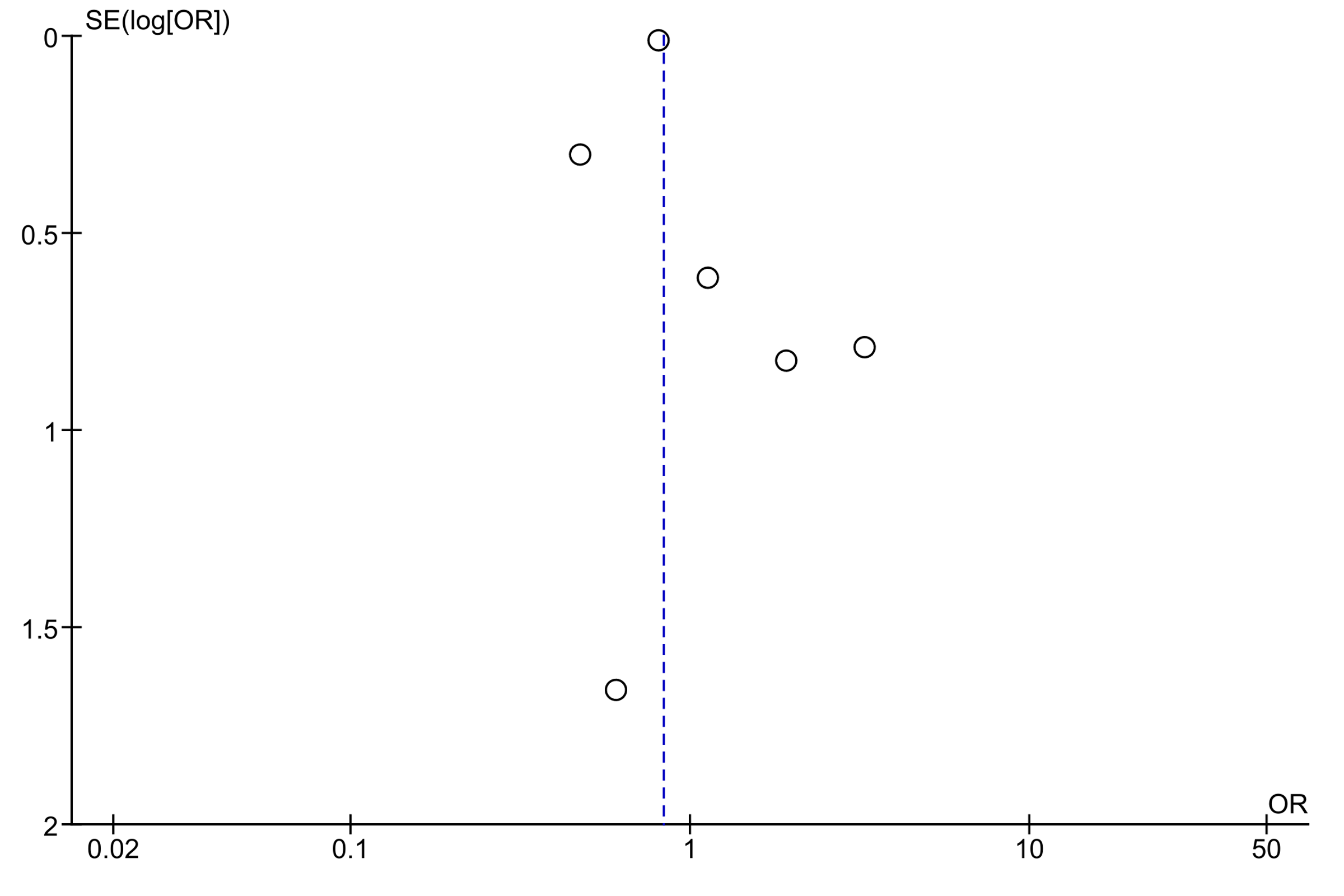


Figure 3 – Readmissions


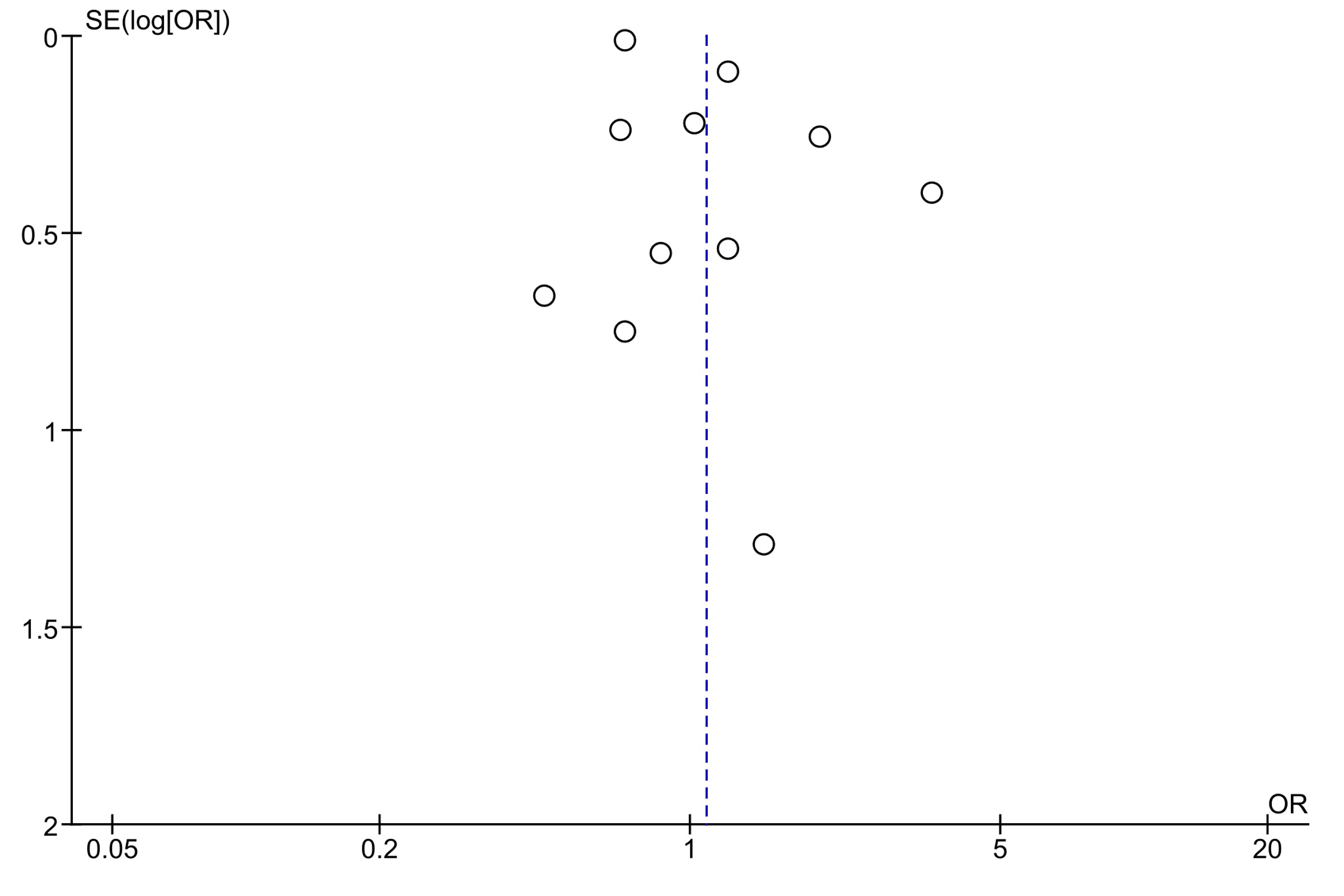


Figure 4 - Post-operative complications


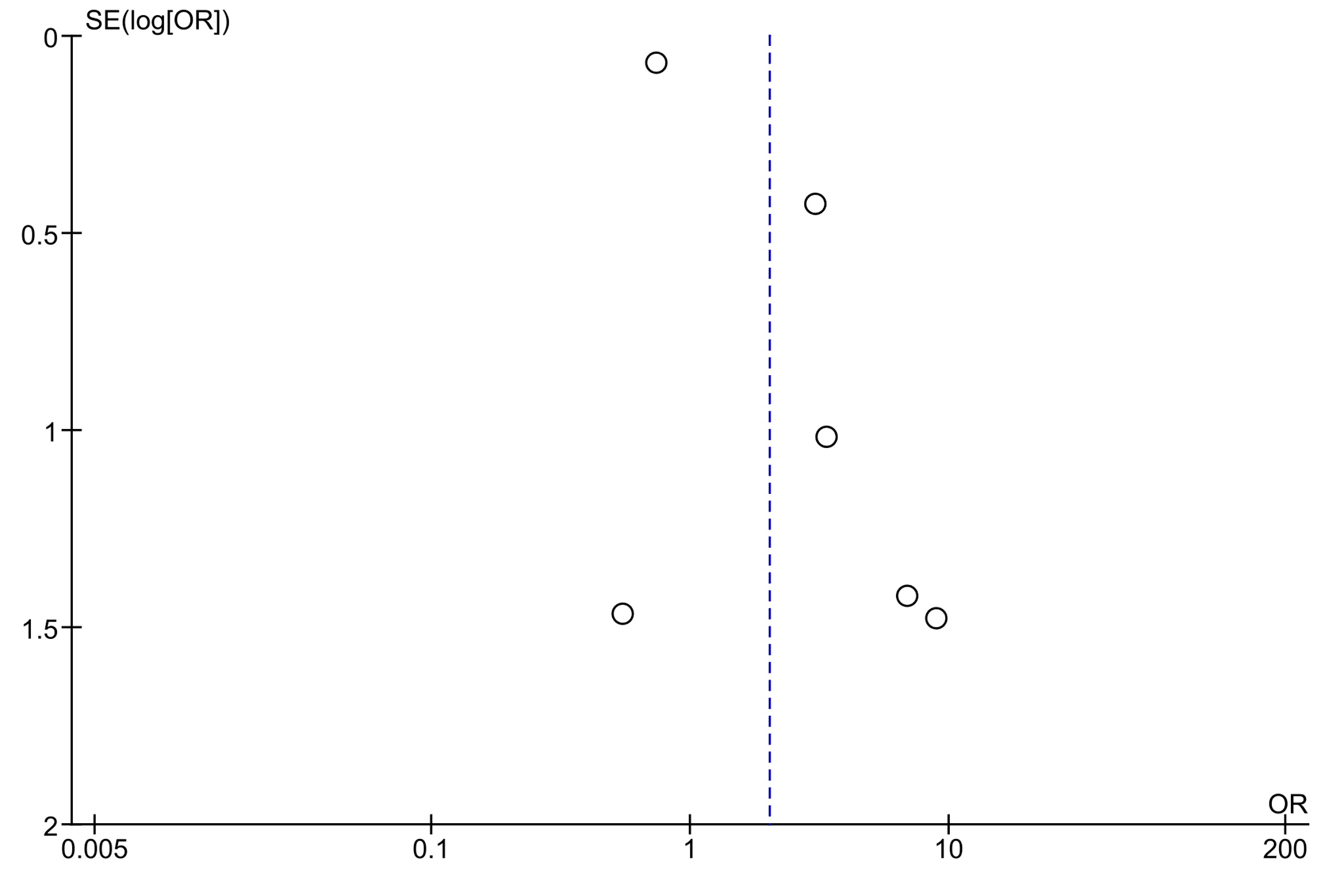


Figure 5 – Mortality


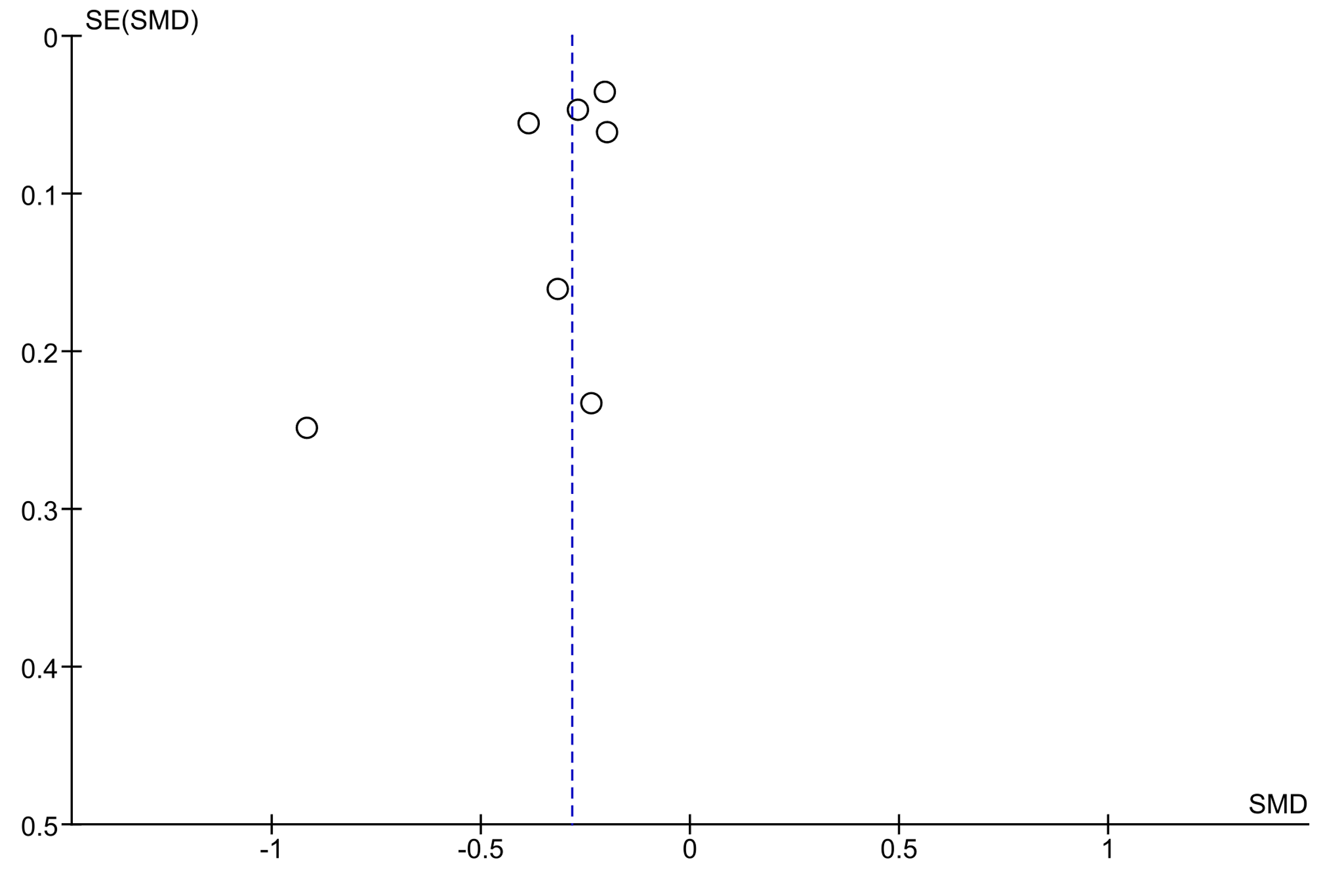


Figure 6 - Cost
